# REDD1 in Clear Cell Renal Cell Carcinoma: Linking Stress Adaptation, Proteostasis and Therapeutic Resistance

**DOI:** 10.3390/cancers18203358

**Published:** 2026-10-17

**Authors:** Sruthi Sureshkumar, Claudia M. Espitia, Fangchao Bi, Rui Xiong, Madison E. Gamble, Natalie L. Hakim, Kevin R. Kelly, Jennifer S. Carew, Steffan T. Nawrocki

**Affiliations:** 1 Division of Translational Medicine, Department of Medicine, University of Arizona, Tucson, AZ 85724, USA; sruthi9830@arizona.edu (S.S.); espitiac@arizona.edu (C.M.E.); madisongamble@arizona.edu (M.E.G.); nhakim@arizona.edu (N.L.H.); jcarew@arizona.edu (J.S.C.); 2 Department of Pharmacology and Toxicology, University of Arizona, Tucson, AZ 85721, USA; bifc@arizona.edu (F.B.); rxiong@pharmacy.arizona.edu (R.X.); 3 Division of Hematology, University of Southern California Keck School of Medicine, Los Angeles, CA 90033, USA; kevin.kelly@med.usc.edu

**Keywords:** REDD1, p97/VCP inhibitors, CB-5339, targeted therapeutics, endoplasmic reticulum stress, unfolded protein response, clear-cell renal cell carcinoma, proteostasis, therapeutic resistance

## Abstract

Clear cell renal cell carcinoma (ccRCC) is the most prevalent form of RCC and is defined by loss of von Hippel–Lindau (VHL) function and dysregulated hypoxia signaling. Hyperactivation of the mTOR pathway is also frequently observed. These factors increase anabolic and protein-synthetic demand leading to proteotoxic stress. To survive under these conditions, ccRCC cells rely on adaptive endoplasmic reticulum (ER) stress responses and autophagy to maintain proteostasis. Regulated in Development and DNA Damage Response 1 (REDD1, also known as DDIT4) is a key stress response protein that integrates hypoxic, metabolic, and ER stress signals to regulate mechanistic target of rapamycin (mTOR) complex 1 (mTORC1) activity and redox homeostasis. REDD1 can either suppress tumor growth through mTOR inhibition or promote survival by facilitating oxidative stress tolerance and autophagy depending on the specific context. In ccRCC, REDD1 expression is driven by VHL loss and HIF stabilization. Yet, mTORC1 activity frequently persists due to dysregulation of upstream repressors such as TSC1/TSC2, direct activating mutations in mTOR, and enhanced growth factor signaling. This paradox underscores the complex interplay between REDD1 and the oncogenic signaling landscape of RCC. Here, we review emerging evidence positioning REDD1 as a pivotal link between hypoxia, mTOR signaling, and ER stress in ccRCC and discuss how therapeutic modulation of this pathway through ER stress inducers, proteasome inhibitors, and p97/valosin-containing protein (VCP) inhibitors may overcome adaptive resistance and improve patient outcomes.

## Simple Summary

Clear cell renal cell carcinoma (ccRCC) is the most common type of kidney cancer and is frequently defined by loss of the tumor-suppressor gene, von Hippel–Lindau (*VHL*). This genetic change disrupts normal oxygen sensing, promotes abnormal growth signaling, and increases protein production, creating substantial stress within cancer cells. To survive, these cells activate protective stress-response pathways. Regulated in Development and DNA Damage Response 1 (REDD1) is a stress-induced protein that helps regulate cell growth, metabolism, and oxidative stress. However, REDD1 can either suppress tumor growth or support tumor-cell survival, depending on the cellular environment. In this review, we discuss the role of REDD1 in clear cell renal cell carcinoma and how targeting REDD1-related stress pathways, including the proteasome and valosin-containing protein (VCP)-dependent protein disposal systems, may provide new strategies to overcome treatment resistance.

## 1. Introduction

Renal cell carcinoma (RCC) is the most common malignancy of the adult kidney and accounts for approximately 85–90% of renal neoplasms in the United States [1]. Clear cell RCC (ccRCC) is the predominant histologic subtype and is responsible for the majority of RCC-related deaths [2]. It accounts for approximately 70–75% of RCC cases, whereas papillary and chromophobe RCC occur considerably less frequently [3]. Although localized disease can often be controlled surgically, approximately 20–30% of patients experience recurrence following nephrectomy [4]. Outcomes decline sharply once metastatic dissemination occurs [5]. The central clinical challenge is therefore not simply controlling the primary tumor but eliminating the stress-tolerant populations that persist during treatment and ultimately drive disease progression.

ccRCC is fundamentally a malignancy of defective oxygen sensing. Somatic mutation of the von Hippel–Lindau (*VHL*) tumor-suppressor gene is detected in approximately 50–60% of sporadic ccRCC cases and represents its defining molecular alteration [6,7,8]. Somatic *VHL* mutations are frequently accompanied by loss of the remaining wild-type allele [6,7,8], while chromosome 3p deletions are observed across localized and advanced disease [9]. Localization of *VHL* to chromosome 3p25 and its recurrent disruption in ccRCC established loss of pVHL function as an early, truncal event in tumor development [10]. This genetic architecture creates a persistent pseudohypoxic state that reshapes tumor metabolism, growth, and stress tolerance. pVHL functions as the substrate-recognition component of an E3 ubiquitin ligase complex. Together with Cullin 2, Elongin B, Elongin C, and Rbx1, pVHL directs selected proteins to the ubiquitin-proteasome system [11,12,13]. Under oxygen-replete conditions, prolyl hydroxylases modify hypoxia-inducible factor (HIF)-1α and HIF2α, permitting their recognition by pVHL [14,15]. The hydroxylated HIFα subunits are subsequently ubiquitinated and degraded by the 26S proteasome [16,17,18]. Hypoxia suppresses the oxygen-dependent activity of the prolyl hydroxylases, allowing HIFα proteins to accumulate [19]. *VHL* loss reproduces this response even when oxygen is available, converting an adaptive hypoxic program into a constitutive oncogenic signal.

Stabilized HIFα translocates to the nucleus and induces genes that support angiogenesis, nutrient acquisition, glycolysis, and survival, including vascular endothelial growth factor (VEGF), platelet-derived growth factor (PDGF), and glucose transporter 1 [20,21]. These effects extend beyond transcriptional adaptation. Activation of VEGF and PDGF receptors stimulates PI3K/AKT signaling and mechanistic target of rapamycin (mTOR) activity [22,23], while mTOR can reinforce HIF expression and function, creating a feed-forward relationship between aberrant oxygen sensing and anabolic growth [24]. The two major HIF isoforms do not contribute equivalently to ccRCC biology. HIF2α promotes tumor growth, angiogenesis, and progression in pVHL-deficient ccRCC [25,26,27] whereas loss or reduced expression of HIF1α is associated with more aggressive disease [28,29]. The balance between these isoforms therefore helps determine the biological consequences of *VHL* inactivation.

The VHL/HIF pathway also intersects with recurrent alterations in the PI3K/AKT/mTOR signaling network. Approximately 28% of ccRCC tumors harbor genetic alterations affecting this pathway [30]. Persistent mTOR activation supports the protein and lipid synthesis required for tumor growth, but it also creates a substantial proteostatic liability. Increased biosynthetic output must be reconciled with limited oxygen and nutrient availability, oxidative stress, and the finite capacity of the protein-folding and degradative machinery [31]. Thus, the same signaling programs that drive ccRCC proliferation also generate cellular stress that must be continuously contained. At the same time, ccRCC has evolved mechanisms that buffer stress. HIF2α-dependent lipid storage also supports ER homeostasis in ccRCC, indicating that lipid accumulation can serve as an adaptive response to metabolic and proteostatic stress [27]. ccRCC cells may therefore exist in a dynamic balance between stress generated by persistent anabolic, metabolic signaling and adaptive mechanisms that preserve ER integrity. This relationship provides a broader framework for understanding ccRCC. These tumors are not sustained solely by angiogenic signaling or uncontrolled proliferation. They are also sustained by an acquired ability to tolerate the metabolic and proteotoxic consequences of those programs. Endoplasmic reticulum (ER) stress responses, the unfolded protein response (UPR), the ubiquitin-proteasome system, and autophagy collectively preserve protein homeostasis and allow ccRCC cells to remain viable under conditions that would otherwise trigger cell death. These adaptive mechanisms are therefore integral components of the malignant phenotype and potential therapeutic vulnerabilities.

**Figure 1:**
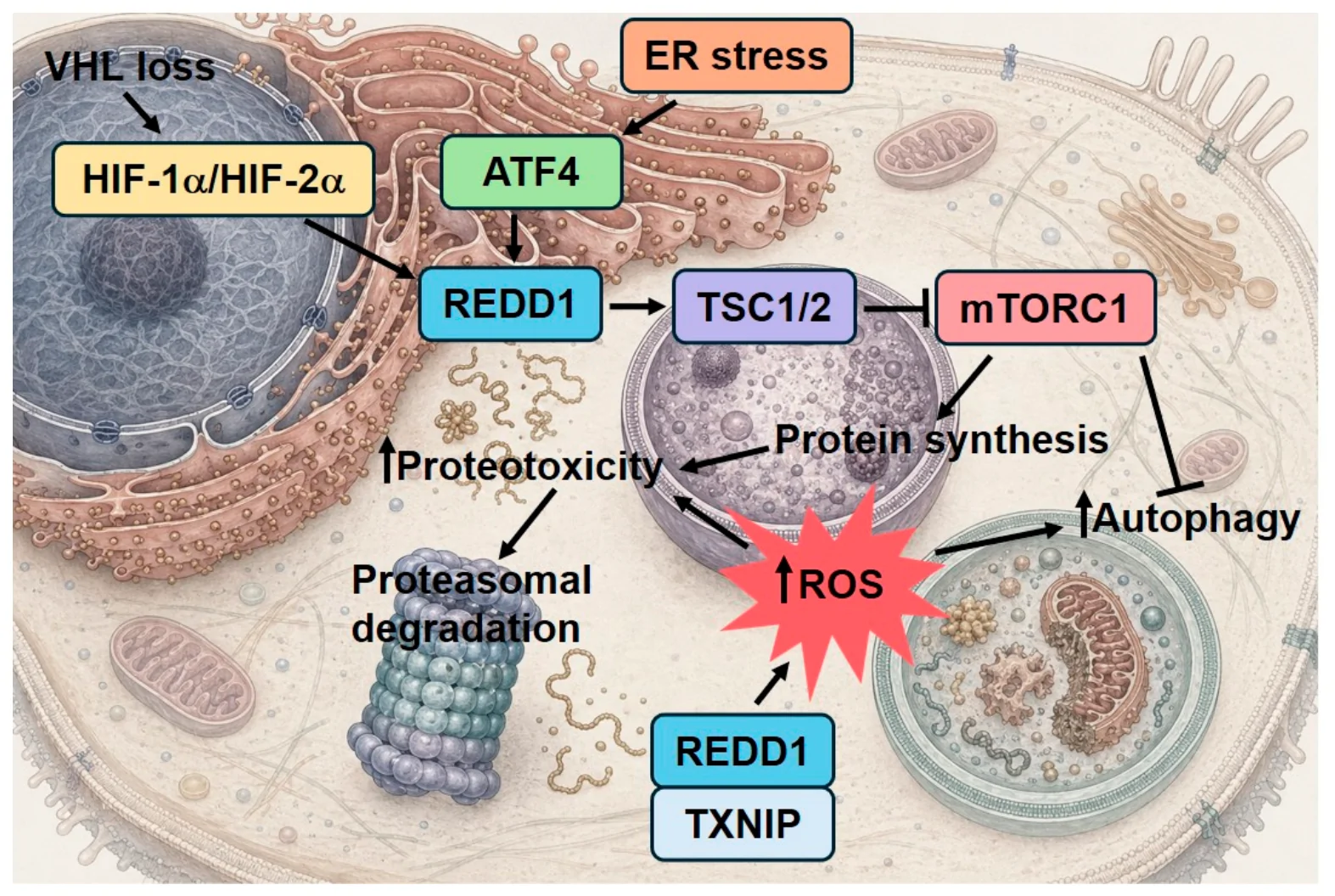
REDD1 integrates VHL/HIF and ER stress signaling with mTORC1, redox control, and proteostasis in ccRCC. In VHL-deficient ccRCC, stabilization of HIF1α/HIF2α and ER stress-mediated activation of ATF4 converge on REDD1. REDD1 signals through TSC1/2 to suppress mTORC1, limiting protein synthesis and the resulting proteotoxic burden while relieving mTORC1-mediated inhibition of autophagy. In parallel, the REDD1–TXNIP axis increases ROS, which further promotes proteotoxic stress and autophagy. Proteasomal degradation and autophagic clearance buffer this stress and preserve protein homeostasis. REDD1 therefore sits at the intersection of pseudohypoxic signaling, anabolic output, oxidative stress, and adaptive protein clearance. The balance among these outputs determines whether tumor cells restore homeostasis or acquire a therapeutically exploitable dependence on proteostasis mechanisms.

Regulated in development and DNA damage response 1 (REDD1, *DDIT4*) lies at the intersection of this oncogenic and stress-adaptive circuitry. Hypoxic, metabolic, oxidative, and ER stress signals converge on REDD1, which can restrain mTORC1-driven anabolism while also influencing redox homeostasis and autophagy. Its biological effect is highly context-dependent. REDD1 can limit tumor growth when its suppression of mTORC1 predominates, but it can also promote survival when its stress-adaptive functions allow malignant cells to withstand metabolic or treatment-induced injury. This distinction is especially important in ccRCC, where *VHL* loss can increase REDD1 expression even as mTORC1 signaling remains active. The resulting state suggests that REDD1 cannot be understood solely as a conventional inhibitor of tumor growth. REDD1 integrates hypoxia, mTOR signaling, ER stress, redox regulation, and autophagy in ccRCC (Figure 1). We first discuss the protein-homeostasis networks that permit cancer cells to survive sustained proteotoxic stress and then examine the regulation and principal functions of REDD1. We subsequently evaluate the context-dependent roles of REDD1 in cancer, its distinctive relationship with the VHL/HIF and mTOR pathways in ccRCC, and the therapeutic implications of disrupting stress adaptation. Finally, we consider strategies that overwhelm proteostasis or disable adaptive recovery, including inhibition of autophagy, the proteasome, and p97/valosin-containing protein (VCP). This framework positions REDD1 not simply as a marker of cellular stress, but as a potential determinant of whether ccRCC cells adapt to stress or are driven beyond the threshold for survival.

## 2. Proteostasis as a Survival Dependency in Cancer

Proteostasis requires continuous coordination among protein synthesis, folding, trafficking, and degradation. This balance is especially difficult to maintain in cancer cells, where oncogenic signaling drives high rates of protein synthesis under conditions of limited oxygen, nutrient deprivation, oxidative injury, and genomic instability. The resulting accumulation of damaged, misfolded, and aggregated proteins places malignant cells under persistent proteotoxic stress [32,33]. Cancer cells compensate by increasing their dependence on otherwise normal stress-response pathways. Because these pathways are not necessarily responsible for tumor initiation but become indispensable for tumor maintenance, this dependence has been termed non-oncogene addiction [32,33]. The heat-shock response, molecular chaperones, ER-associated degradation (ERAD), the ubiquitin-proteasome system, and autophagy collectively buffer proteotoxic injury and allow cancer cells to maintain the protein quality required for continued survival [33,34,35].

The ER occupies a central position within the proteostasis network. Secretory and membrane proteins enter the ER for folding, post-translational modification, and quality-control assessment before being transported to their final destinations [36,37,38]. Conditions that exceed the folding capacity of the ER cause unfolded or misfolded proteins to accumulate within its lumen, producing ER stress. Cancer cells frequently operate close to this proteostatic limit and therefore rely heavily on the UPR to prevent an otherwise lethal collapse in protein homeostasis [36,37,38]. The UPR is initiated by three principal ER stress sensors: protein kinase R-like ER kinase (PERK), inositol-requiring enzyme 1α (IRE1α), and activating transcription factor 6 (ATF6). Under basal conditions, the ER chaperone glucose-regulated protein 78 (GRP78/BiP) associates with these sensors and maintains them in an inactive state [39]. This buffering capacity can support malignant growth by allowing cancer cells to accommodate increased protein-folding demand [34]. As unfolded proteins accumulate, BiP is recruited away from the stress sensors, permitting their activation [40]. The three UPR branches then engage complementary mechanisms that reduce the incoming protein burden, expand the folding capacity of the ER, and remove proteins that cannot be repaired.

Upon ER stress, ATF6 translocates to the Golgi, where it is proteolytically activated before entering the nucleus and inducing genes encoding molecular chaperones, ER quality-control proteins, and X-box binding protein 1 (XBP1) [41]. Activated IRE1α functions as both a kinase and an endoribonuclease. Its RNase domain removes a 26-nucleotide intron from *XBP1* mRNA, generating the transcriptionally active XBP1s isoform [42]. XBP1s subsequently increases the expression of chaperones, lipid-biosynthetic enzymes, and ERAD components that expand ER capacity and facilitate the clearance of terminally misfolded proteins [41]. Together, the ATF6 and IRE1α/XBP1 pathways remodel the ER to accommodate sustained biosynthetic and secretory demand. PERK controls the translational and cell-fate components of the UPR. Activated PERK phosphorylates eukaryotic translation initiation factor 2α (eIF2α), producing a rapid reduction in global protein synthesis while selectively favoring translation of activating transcription factor 4 (ATF4) [43]. This response initially protects the cell by limiting the influx of newly synthesized proteins into the ER and activating genes involved in amino acid metabolism, antioxidant defense, and protein quality control. ATF4 also induces autophagy-related genes, including *SQSTM1/p62*, *ATG5*, *ATG7*, and *ATG10*, thereby linking ER stress to lysosomal clearance of damaged proteins and organelles [44]. Autophagy is predominantly adaptive during the early response to ER stress because it removes potentially toxic material and recycles intracellular substrates. Its biological effect, however, depends on the intensity and duration of the stress. When ER injury cannot be resolved, sustained autophagic signaling can contribute to cell death, including activation of FADD- and caspase-8-dependent apoptotic pathways [45]. The relevant therapeutic question is therefore not whether autophagy is intrinsically protective or cytotoxic. It is whether autophagic capacity remains sufficient to restore homeostasis or has become part of a terminal stress response.

The PERK/eIF2α/ATF4 pathway also determines whether the UPR remains adaptive by inducing stress-response proteins that regulate metabolism, redox homeostasis, and apoptosis. These include C/EBP homologous protein (CHOP) [46] and REDD1 [47]. During persistent ER stress, CHOP suppresses the prosurvival protein BCL-2, disrupts redox balance, and increases mitochondrial susceptibility to apoptosis [45,48]. CHOP can also promote thioredoxin-interacting protein (TXNIP) dependent mitochondrial signaling and thereby enhance ROS production, linking unresolved ER stress to oxidative injury [49]. TXNIP, in turn, interacts with and stabilizes REDD1, thereby enhancing REDD1-dependent suppression of mTOR signaling [50]. This relationship places REDD1 at a mechanistically important intersection between the UPR, oxidative stress, and control of anabolic growth.

## 3. Molecular Regulation and Functional Roles of REDD1

### 3.1. REDD1 as a Rapidly Inducible Stress Integrator

REDD1, encoded by *DDIT4* and also known as RTP801 or Dig2, is a stress-responsive protein induced by diverse environmental and intracellular signals [51,52,53]. The human *DDIT4* gene is located on chromosome 10 and encodes a 232-amino-acid protein with a molecular mass of approximately 25 kDa [53,54]. REDD1 is an acidic, serine-rich protein that lacks an established catalytic domain or intrinsic enzymatic activity [53,54,55]. Its low basal abundance and short half-life, reported to range from approximately 5 to 20 min, permit REDD1 levels to change rapidly as stress conditions evolve. These features distinguish REDD1 from a conventional signaling enzyme and instead position it as a transient regulator whose activity is determined by the timing, magnitude, and duration of its expression. REDD1 was initially identified as the hypoxia-responsive gene RTP801 in models of ischemic injury [51,52,53]. Although *DDIT4* transcripts were detectable in multiple adult tissues, REDD1 protein abundance was generally low under basal conditions and increased markedly following hypoxic stress [52]. Independently, REDD1 was identified as a transcriptional target of p53 following DNA damage and of p63 during epithelial differentiation [53]. The range of signals capable of inducing REDD1 has since expanded considerably. REDD1 expression is responsive to cellular stress, hormonal signaling, and metabolic imbalance [52,54]. Direct stress inputs include hypoxia, reactive oxygen species (ROS) [56], and DNA damage [53]. REDD1 is also induced following inhibition of fatty acid synthesis and glucocorticoid exposure [57,58], insulin signaling [59,60], catecholamine stimulation [61], and hyperglycemic stress [62]. Vitamin D can regulate REDD1-dependent metabolic signaling [63,64], whereas amino acid deprivation, fasting, and other disturbances in nutrient availability activate REDD1 through integrated metabolic stress pathways [62,65,66]. REDD1 is therefore not a selective marker of any single insult. It is a convergence point through which diverse physiological and pathological conditions can restrain anabolic activity and reprogram cellular stress responses.

Several transcriptional programs, including ER stress and amino acid deprivation, engage an ATF4–REDD1/TXNIP module that increases REDD1 expression and stabilizes the protein [50,67]. HIF1α, p53, CCAAT/enhancer-binding proteins, and Elk-1 can also promote *DDIT4* transcription in response to distinct forms of cellular injury [52,53,68]. Ataxia-telangiectasia mutated (ATM) kinase provides an additional link between hypoxic and genotoxic stress. Under hypoxia, ATM-dependent phosphorylation of HIF1α at serine 696 enhances HIF1α transcriptional activity and REDD1 induction [69]. ATM also reinforces p53 signaling following DNA damage [70], providing another route to p53-dependent induction of REDD1 [66]. These inputs create a highly integrated regulatory network in which oxygen availability, nutrient status, oxidative stress, and genomic damage can all influence REDD1 expression. REDD1 abundance is also tightly controlled after transcription. One study identified REDD1 as a substrate of a CUL4A–DDB1–ROC1–β-TRCP E3 ubiquitin ligase complex. In this model, GSK3β-dependent phosphorylation promotes REDD1 ubiquitination and proteasomal degradation, allowing mTOR signaling to recover as cells emerge from hypoxic stress [55]. A subsequent study reported a different regulatory mechanism. In that analysis, activation of mTORC1 increased REDD1 protein stability, whereas pharmacologic or genetic inhibition of mTORC1 accelerated its proteasome-dependent turnover. The investigators did not identify a requirement for CUL4A, HUWE1, or other Cullin-RING E3 ligases [71]. These findings support an mTORC1–REDD1 negative-feedback circuit in which active mTORC1 stabilizes an inhibitor that can subsequently restrain its signaling.

The differing models of REDD1 degradation should not be collapsed into a single definitive mechanism. Instead, they suggest that REDD1 turnover varies with cell type, stress context, and whether cells are entering, sustaining, or recovering from stress. The specific E3 ligase responsible for REDD1 degradation may likewise differ across biological settings. What is clear is that rapid proteasomal turnover is integral to REDD1 function. Transcriptional induction generates a rapid REDD1 pulse, whereas post-translational degradation limits its duration and permits restoration of growth signaling once the stress has resolved. REDD1 is therefore regulated as a dynamic stress rheostat rather than a static on/off switch. HIF, ATF4, and p53 determine when REDD1 is induced, while proteasomal turnover and feedback from mTORC1 determine how long its activity persists. This temporal regulation is central to its context-dependent biology. A short REDD1 pulse can transiently reduce anabolic demand and facilitate recovery, whereas sustained expression can produce prolonged mTOR suppression, alter redox homeostasis, and reshape autophagic dependence.

### 3.2. REDD1 Restrains mTORC1 Through the TSC-Rheb Axis

mTOR is a serine/threonine kinase that integrates growth-factor signaling, nutrient availability, energy status, and cellular stress to determine whether cells engage anabolic growth or conserve resources [72,73]. mTOR functions within two structurally and functionally distinct complexes. mTORC1 contains the defining scaffold protein RAPTOR, whereas mTOR complex 2 (mTORC2) contains RICTOR [74,75,76]. mTORC1 promotes protein and lipid synthesis while suppressing autophagy, thereby coupling nutrient sufficiency to biomass accumulation. By contrast, mTORC2 regulates AKT signaling, cellular survival, metabolism, and cytoskeletal organization [77]. Because REDD1 primarily regulates mTORC1, its induction provides a direct mechanism through which stress can override growth-promoting signals and rapidly reduce anabolic demand. Two of the best-characterized mTORC1 substrates are ribosomal protein S6 kinase 1 (S6K1) and eukaryotic translation initiation factor 4E-binding protein 1 (4E-BP1) [78]. Activated S6K1 phosphorylates ribosomal protein S6 and additional components of the translational machinery, promoting cell growth and protein synthesis [79]. Hypophosphorylated 4E-BP1 binds eIF4E and prevents assembly of the cap-dependent translation-initiation complex. mTORC1-dependent phosphorylation releases 4E-BP1 from eIF4E, permitting translation to proceed [80]. Accordingly, inhibition of mTORC1 by REDD1 reduces S6K1 and 4E-BP1 phosphorylation and rapidly constrains the translational output that contributes to proteotoxic stress.

**Figure 2:**
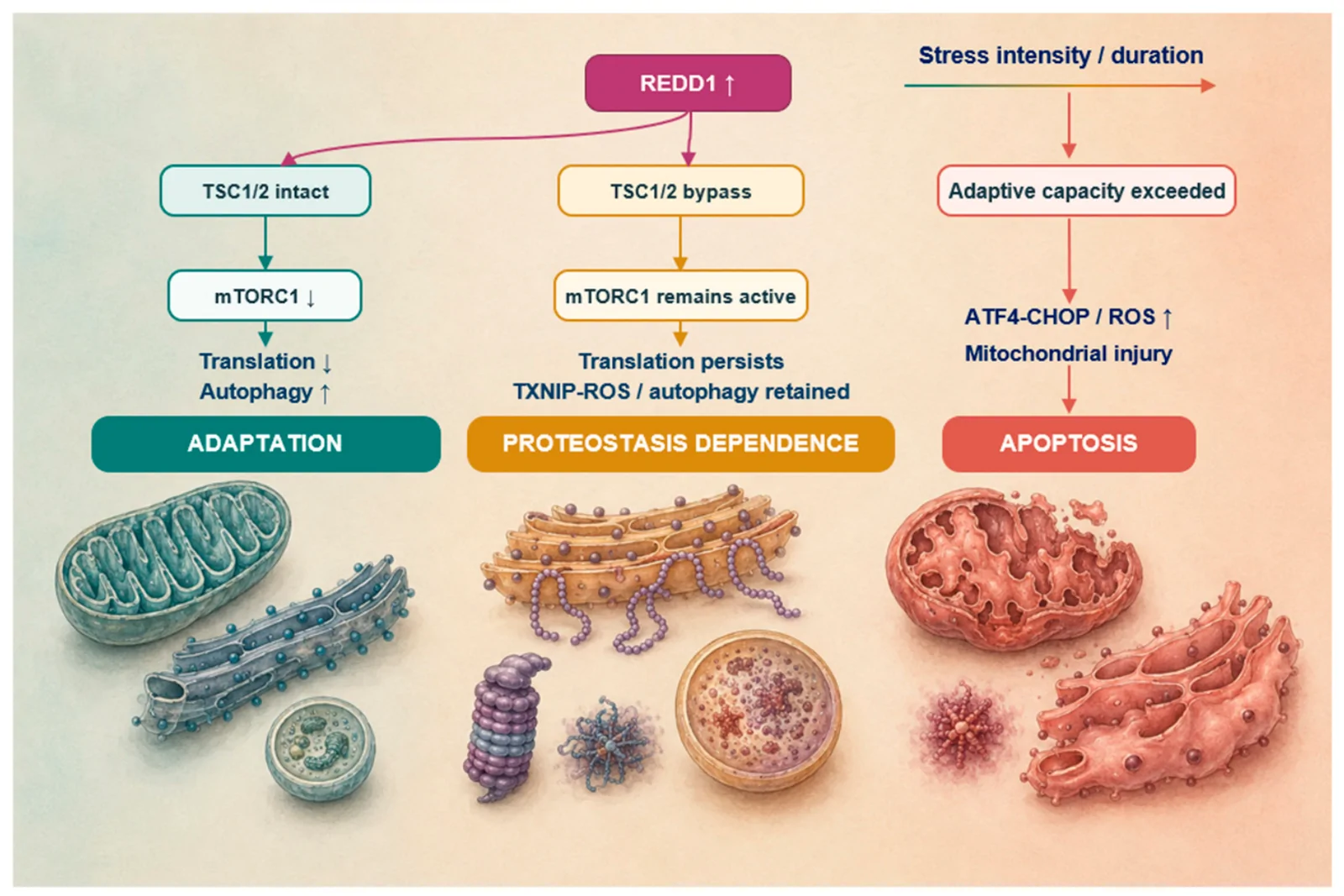
REDD1 acts as a context-dependent stress rheostat that determines adaptation, proteostasis dependence, or apoptosis. When the TSC1/2 checkpoint is intact, REDD1 induction suppresses mTORC1, reduces translation, and increases autophagy, thereby promoting cellular adaptation. When TSC1/2 control is bypassed, mTORC1 activity and translation persist despite REDD1 induction, while TXNIP–ROS signaling and autophagy are retained. Persistent anabolic demand must therefore be sustained by adaptive clearance pathways, creating a state of heightened proteostasis dependence. As stress intensity or duration increases, adaptive capacity is eventually exceeded. Sustained ATF4–CHOP and ROS signaling then promotes mitochondrial injury and apoptosis. REDD1 abundance alone does not predict biological outcome; its functional consequences are determined by TSC1/2 competence, coupling to mTORC1, and the magnitude and duration of cellular stress.

The TSC1/TSC2 tumor-suppressor complex is the principal signaling gate between REDD1 and mTORC1. TSC1, also known as hamartin, stabilizes TSC2 and protects it from degradation, while growth-factor-dependent phosphorylation of TSC2 by AKT suppresses its activity and alters its subcellular distribution [81,82]. TSC2 contains a GTPase-activating protein domain that accelerates the conversion of Ras homolog enriched in brain (Rheb) from its active GTP-bound state to its inactive GDP-bound state [83,84]. Rheb-GTP directly activates mTORC1. Thus, an active TSC1/TSC2 complex reduces Rheb-GTP loading and suppresses mTORC1, whereas AKT-mediated inhibition of TSC2 permits Rheb-dependent mTORC1 activation. Early work proposed that REDD1 activates the TSC complex by altering the interaction between TSC2 and 14-3-3 proteins. In this model, growth-factor signaling promotes AKT-dependent phosphorylation of TSC2 and its association with inhibitory 14-3-3 proteins. Stress-induced REDD1 was proposed to bind 14-3-3, displace TSC2 from the complex, and restore TSC2-dependent inhibition of Rheb and mTORC1 [54]. This model provided an attractive explanation for how a protein lacking enzymatic activity could transmit an inhibitory signal to mTORC1 (Figure 2).

Subsequent structural studies indicated that this mechanism is more complex. The REDD1 crystal structure revealed a novel α/β-fold containing two interdependent regions required for mTORC1 inhibition. Structure-based docking and biochemical analyses did not support direct binding between REDD1 and 14-3-3. Instead, mutational mapping identified a conserved surface hotspot required for REDD1 activity and likely to recruit one or more unidentified effector proteins. Thus, REDD1 may influence the TSC2–14-3-3 complex indirectly or may operate through additional intermediates. Importantly, the structural findings did not challenge the requirement for TSC1/TSC2. REDD1-mediated inhibition of mTORC1 remained dependent on an intact TSC complex [54,85,86]. Genetic evidence strongly supports this TSC dependence. Hypoxia-induced REDD1 suppresses mTORC1 signaling in wild-type cells but fails to do so efficiently in TSC2-deficient mouse embryonic fibroblasts [54,85]. REDD1 therefore acts upstream of TSC2 rather than directly inhibiting the mTORC1 kinase complex. This distinction has important implications for cancer. REDD1 can be highly expressed yet functionally unable to suppress mTORC1 when the TSC–Rheb signaling gate has been disrupted. REDD1 abundance alone consequently cannot be used as a surrogate for REDD1 activity.

A second mechanistic model links REDD1 to site-specific regulation of AKT. REDD1 associates with AKT and the catalytic subunit of protein phosphatase 2A (PP2A), promoting PP2A-dependent dephosphorylation of AKT at Thr308 [87]. Loss of Thr308 phosphorylation reduces AKT activity toward TSC2, decreases inhibitory TSC2 phosphorylation, and lowers the abundance of Rheb-GTP. The result is TSC2-dependent suppression of mTORC1. Notably, this effect is site selective: REDD1 can reduce AKT Thr308 phosphorylation without producing an equivalent reduction at Ser473. REDD1 may therefore alter AKT substrate activity rather than fully inactivating AKT. The 14-3-3 and PP2A–AKT models are not necessarily mutually exclusive. Both converge on preservation of TSC2 activity and reduction of Rheb-GTP loading, and their relative importance may vary with cell type, stressor, and signaling context. REDD1 may function as a scaffold that organizes different regulatory proteins rather than acting through a single invariant binding partner. This interpretation is consistent with its lack of catalytic activity and with the context-dependent effects attributed to REDD1 across normal and malignant tissues. These findings establish an important principle: induction of REDD1 does not automatically produce mTORC1 inhibition. The functional output depends on the integrity of TSC1/TSC2, the activation state of AKT and Rheb, and the availability of REDD1-associated effectors. When this regulatory network remains intact, REDD1 reduces protein synthesis, lowers energetic demand, and facilitates adaptation to stress. When the TSC–Rheb axis is genetically or functionally uncoupled, mTORC1 can remain active despite abundant REDD1. Under those conditions, the non-mTOR functions of REDD1, particularly its effects on oxidative signaling and autophagy, may become the dominant determinants of cell fate.

### 3.3. REDD1 Exerts Compartment-Specific Control of Redox Homeostasis

REDD1 has been described as both a pro-oxidant and an antioxidant, and the available evidence indicates that neither designation is universally correct. Its redox effects depend on its binding partner, subcellular localization, and the nature of the initiating stress. For example, *TP63*-null fibroblasts with reduced REDD1 expression accumulate ROS, consistent with an antioxidant function [53]. Conversely, REDD1 induction in neuronal models of ischemic stress has been associated with ROS accumulation and cell death [52]. These apparently divergent findings reflect the ability of REDD1 to regulate distinct cytosolic and mitochondrial redox circuits rather than a single directional effect on ROS.

The best-defined pro-oxidant mechanism involves TXNIP. Thioredoxin is a major cellular antioxidant that reduces oxidized proteins and is subsequently regenerated by thioredoxin reductase using NADPH. TXNIP binds reduced thioredoxin through a disulfide-exchange reaction and inhibits its antioxidant activity [88,89,90]. This interaction compromises the thioredoxin–thioredoxin reductase–NADPH system, reduces the capacity to neutralize ROS, and shifts the intracellular environment toward oxidation. REDD1 forms a stress-inducible complex with TXNIP that stabilizes both proteins and generates a localized pro-oxidant signal. Formation of this complex is necessary for efficient TXNIP-dependent ROS production, as the pro-oxidant activity of TXNIP is attenuated in REDD1-deficient cells [91]. The REDD1–TXNIP complex also links oxidative signaling to autophagy. By inhibiting thioredoxin, the complex promotes oxidation of the cysteine protease ATG4B, thereby limiting ATG4B-mediated delipidation of LC3 and supporting autophagosome formation during stress [91]. However, this mechanism has been demonstrated in U2OS human osteosarcoma cells and REDD1-null and TXNIP-null mouse embryonic fibroblasts (MEFs). Although the study highlights the relevance of REDD1 in redox biology and autophagy in these experimental systems, it has not directly established the REDD1–TXNIP complex mechanism in ccRCC. In this setting, ROS are not simply nonspecific damaging molecules, but they function as spatially restricted signals that modify the autophagy machinery. Whether this response promotes adaptation or cell death depends on the magnitude and duration of the oxidative signal. REDD1/TXNIP signaling has been implicated in hyperglycemia-associated stress and oxidative apoptosis [62], while disruption of the REDD1–TXNIP axis reduces oxidative injury and apoptosis in endothelial cells [92]. A controlled increase in ROS can facilitate autophagy and metabolic adaptation. Sustained thioredoxin inhibition, however, can exceed antioxidant capacity, produce mitochondrial injury, and engage apoptosis. The same REDD1–TXNIP complex can therefore support short-term stress tolerance or contribute to irreversible oxidative damage.

REDD1 can also amplify ROS through an AKT/GSK3-dependent feedback circuit. Under hyperglycemic conditions, REDD1 expression, mitochondrial membrane potential, and ROS production increase in parallel. These responses are markedly attenuated in REDD1-deficient cells and tissues [93]. Mechanistically, REDD1 promotes PP2A-dependent inhibition of AKT, reducing inhibitory phosphorylation of GSK3β and thereby increasing GSK3β activity. Expression of dominant-negative AKT or constitutively active GSK3β restores mitochondrial hyperpolarization and ROS production in REDD1-deficient cells, placing the AKT/GSK3 pathway downstream of REDD1 [93]. ROS can, in turn, increase REDD1 expression, creating a feed-forward loop that sustains oxidative stress. A distinct pool of REDD1 regulates mitochondrial function at mitochondria-associated ER membranes (MAMs). These specialized contact sites coordinate calcium transfer, lipid metabolism, mitochondrial respiration, and anabolic signaling. At MAMs, the inositol 1,4,5-trisphosphate receptor (IP3R) on the ER communicates with the voltage-dependent anion channel (VDAC) on the outer mitochondrial membrane through the molecular chaperone GRP75. This complex facilitates calcium transfer from the ER to mitochondria, supporting oxidative metabolism and ATP production [94,95,96]. AKT and mTOR signaling also operate at these contact sites, coupling mitochondrial energy generation to anabolic processes within the ER [94]. REDD1 localizes to MAMs and interacts with components of the IP3R–GRP75–VDAC complex. Increased REDD1 expression reduces the extent of ER–mitochondrial contacts, decreases oxygen consumption and ATP production, and limits energy-intensive anabolic processes [97]. These REDD1-dependent effects were established in primary human myoblasts and mouse skeletal-muscle models. Whether REDD1 similarly regulates MAM integrity and mitochondrial function in ccRCC remains unknown. In this context, REDD1 functions as a metabolic brake. By reducing mitochondrial respiratory activity, REDD1 can also limit mitochondrial ROS generation. REDD1-mediated disruption of MAMs should therefore not be interpreted automatically as a pro-oxidant event. Indeed, the effects of altered MAM integrity vary by tissue and stress context. GRP75 depletion in adipocytes, for example, disrupts MAM organization, reduces manganese superoxide dismutase expression, and increases intracellular ROS. The redox consequences of MAM remodeling therefore depend on how a particular cell balances calcium transfer, respiratory activity, and antioxidant capacity.

A direct antioxidant function for REDD1 is also supported by studies of HIF regulation. Loss of REDD1 increases mitochondrial ROS, stabilizes HIF1α, and enhances HIF-dependent transcription and glycolytic metabolism [98]. REDD1-deficient MEFs consequently gain a selective growth advantage under hypoxia and display increased tumorigenicity. Antioxidant treatment or HIF1α depletion reverses this phenotype, demonstrating that ROS-dependent HIF activation is functionally responsible for the increased tumor growth. In this setting, REDD1 completes a negative-feedback loop in which HIF1α induces REDD1, and REDD1 subsequently limits mitochondrial ROS and destabilizes HIF1α. This antioxidant activity contributes directly to tumor suppression. The redox biology of REDD1 can therefore be organized according to compartment and interacting partner. Cytosolic REDD1 complexed with TXNIP inhibits thioredoxin and promotes ROS-dependent autophagy or apoptosis. REDD1-mediated inhibition of AKT can amplify mitochondrial ROS through GSK3-dependent signaling. In contrast, MAM-associated REDD1 reduces mitochondrial respiration and can limit ROS production, while REDD1-dependent suppression of mitochondrial ROS restrains HIF1α signaling and tumorigenesis. The net effect is determined by which REDD1 pool predominates and whether the resulting ROS remain within an adaptive signaling range or cross the threshold for oxidative injury. This context dependence is particularly relevant to cancer. Moderate REDD1-dependent ROS production may sustain autophagy and permit survival under metabolic stress, whereas prolonged oxidative amplification may trigger apoptosis. Conversely, the antioxidant activity of REDD1 may suppress ROS-driven tumorigenic signaling while simultaneously protecting established cancer cells from lethal oxidative injury. REDD1 is therefore neither primarily pro-oxidant nor primarily antioxidant. It is a compartmentalized regulator of redox signaling whose biological effect is determined by the stress-adaptive state of the cell.

### 3.4. REDD1 Functions as a Context-Dependent Tumor Suppressor and Promoter

Initial observations supported classification of REDD1 as a tumor suppressor. Reduced REDD1 expression was identified in subsets of human breast and prostate cancers relative to nonmalignant epithelium, consistent with the loss of an endogenous restraint on mTORC1 signaling [54]. This interpretation is reinforced by the broader consequences of disrupting the TSC–Rheb pathway. TSC1- or TSC2-mutant hepatocellular carcinomas display hyperactive mTOR signaling and more aggressive tumor behavior, suggesting the upregulation of mTORC1 mediates the proliferation of tumor cells [99]. Although these findings do not directly establish REDD1 loss as a driver of hepatocellular carcinoma, they demonstrate that the antitumor effect of REDD1 ultimately depends on a functional TSC complex. When this downstream signaling gate is disabled, REDD1 induction is unlikely to produce durable mTORC1 suppression. Several pharmacologic studies further support a tumor-suppressive role for acute REDD1 induction. In breast cancer cells, melatonin enhances arsenic trioxide-induced ROS generation and apoptosis through sustained upregulation of REDD1. Zoledronic acid similarly activates the PERK–eIF2α–CHOP arm of the ER stress response, induces REDD1, suppresses AKT/mTOR signaling, and reduces breast cancer cell growth. Analysis of human breast tumors also demonstrated lower REDD1 expression in malignant tissues than in benign papilloma controls. Reduced REDD1 was associated with persistent mTOR activity and HIF1α stabilization, particularly in HER2-overexpressing and triple-negative disease [100,101,102]. Together, these findings support a model in which loss of REDD1 removes a stress-responsive barrier to anabolic growth and hypoxic signaling. The p53–REDD1 axis also contributes to pharmacologic growth arrest. In prostate cancer cells, metformin induces REDD1 in a p53-dependent but AMPK-independent manner. REDD1 depletion prevents metformin-mediated mTOR inhibition, reverses cell-cycle arrest, and protects against suppression of cellular transformation [103]. REDD1 is therefore not merely a correlative marker of metformin exposure in this setting. It is a functional mediator of the antiproliferative response. These studies collectively indicate that acute induction of REDD1 can be therapeutically beneficial when it produces sustained mTORC1 inhibition, cell-cycle arrest, or oxidative injury.

However, increased REDD1 expression is not uniformly associated with tumor suppression. High cytoplasmic REDD1 expression in ovarian carcinoma correlates with advanced-stage disease, ascites, incomplete response to chemotherapy, and inferior survival. Functional studies further associated REDD1 with increased migration and invasion, suggesting that chronically elevated REDD1 can support an aggressive tumor phenotype. In bladder urothelial carcinoma, REDD1 promotes proliferation and autophagy while limiting apoptosis. REDD1 inhibition, either directly or through miR-22, suppresses the REDD1–EEF2K–autophagy axis and sensitizes tumor cells to paclitaxel in vitro and in vivo. This finding is especially important because AKT/mTOR signaling recovered rapidly following REDD1 manipulation, whereas the autophagy and drug-sensitivity phenotypes persisted. The tumor-supportive activity of REDD1 therefore cannot always be explained by sustained mTORC1 inhibition [104,105,106,107]. A similar association has been observed in oral squamous cell carcinoma, where REDD1 is elevated relative to adjacent mucosa and correlates with advanced stage, poor differentiation, lymphatic metastasis, recurrence, and reduced survival. REDD1 expression also correlates positively with microvessel density, linking it to the angiogenic phenotype of these tumors [108]. Collectively, these findings challenge the assumption that an endogenous mTORC1 inhibitor must function as a tumor suppressor. Under chronic hypoxic or metabolic stress, REDD1 may instead create a low-anabolic, autophagy-competent state that favors tumor persistence and therapeutic resistance.

High *DDIT4* expression has been associated with inferior outcomes in acute myeloid leukemia, breast cancer, glioblastoma, colon cancer, skin cancer, and lung cancer, but with improved outcomes in gastric cancer [109]. No significant prognostic relationship was detected in ovarian cancer in the same transcriptomic analysis. The lack of an ovarian association at the mRNA level despite the adverse relationship observed for cytoplasmic REDD1 protein illustrates the limitations of interpreting bulk *DDIT4* expression in isolation. Transcript abundance does not capture protein stability, subcellular localization, signaling competence, or whether REDD1 is expressed by tumor cells, stromal cells, or stress-associated subpopulations. The duration of REDD1 expression provides one framework for reconciling these findings. Transient induction can rapidly suppress mTORC1, reduce proliferation, and facilitate recovery from an acute stress. If the insult remains unresolved, sustained REDD1 can amplify ROS and promote apoptosis. In an established tumor that has adapted to chronic REDD1 expression, however, the consequences may be fundamentally different. Reduced translation lowers ATP demand, autophagy preserves metabolic substrates, and attenuation of growth-factor signaling can produce a slow-cycling state that is less vulnerable to therapy. REDD1 can therefore suppress tumor initiation or acute proliferation while supporting the survival of an already established, stress-adapted malignancy. Importantly, much of the evidence defining the functions of REDD1 comes from malignancies other than ccRCC. These studies therefore provide the mechanistic framework for interpreting REDD1 biology, rather than direct evidence that these functions operate similarly in ccRCC.

REDD1 can restrain mTORC1 only when the TSC–Rheb signaling axis remains responsive. Tumors with downstream pathway activation may retain REDD1-mediated effects on redox signaling, mitochondrial metabolism, and autophagy while becoming resistant to its growth-suppressive output. This uncoupling creates a particularly unfavorable state in which cancer cells maintain anabolic signaling, while simultaneously exploiting REDD1-dependent stress adaptation. REDD1 should therefore be evaluated as a functional state rather than classified categorically as an oncogene or tumor suppressor. The relevant questions are whether REDD1 effectively suppresses mTORC1, promotes adaptive or terminal autophagy, maintains tolerable redox output, and is transiently or chronically expressed (Table 1). This framework is essential for interpreting REDD1 in ccRCC, where constitutive hypoxic signaling, mTOR activation, ER stress, and autophagy converge within the same tumor cells.

## 4. ER Stress and REDD1 Create an Adaptive Vulnerability in ccRCC

The kidney is continuously exposed to metabolic, oxidative, and proteotoxic stress, and chronic renal injury has been mechanistically and epidemiologically linked to renal carcinogenesis [110]. Diabetic nephropathy provides one nonmalignant example of how sustained metabolic injury engages the ER stress response in renal tissue. Activation of the UPR is observed across experimental and clinical studies of diabetic kidney disease while hyperglycemia promotes oxidative stress, inflammation, mitochondrial dysfunction, and renal epithelial injury [111]. Aberrant mTORC1 and insulin-like growth factor signaling further contribute to the pathological response of the kidney to metabolic stress [112,113]. In ccRCC, this stress state is directly linked to its defining genetic lesion. Experimental inactivation of *VHL* in nonmalignant kidney epithelial cells increases protein synthesis, ROS, and expression of ER stress markers, including BiP, XBP1s, phosphorylated IRE1α, and phosphorylated PERK. IRE1α kinase activity is required for downstream activation of JNK and NF-κB and for the recruitment of macrophages to *VHL*-deficient cells. Pharmacologic reduction of ER stress or inhibition of IRE1α suppresses this inflammatory response [114]. Thus, *VHL* loss does more than stabilize HIF and promote angiogenesis. It creates a proteostatic and inflammatory state that can shape the earliest stages of ccRCC development.

Autophagy is a major component of the adaptive response to this stress. ER stress and mTOR signaling converge on the autophagy machinery, which removes damaged proteins and organelles while recycling metabolic substrates. In RCC, autophagy has been implicated in tumor initiation, progression, survival, and resistance to therapy [115,116,117]. Its effect is strongly context-dependent: basal or treatment-induced autophagy can protect RCC cells from metabolic injury, whereas excessive or incomplete autophagy can contribute to cell death. In established ccRCC, however, the predominant consequence is often preservation of tumor-cell fitness under hypoxia, nutrient limitation, and therapeutic stress. Sunitinib induces ER stress in RCC cells but can simultaneously activate NF-κB and stimulate the expression of proinflammatory, protumorigenic cytokines [118]. This response promotes a refractory phenotype rather than simply increasing cell death. The finding illustrates a central limitation of stress-inducing therapy: increasing ER stress is not sufficient if tumor cells retain the capacity to mount an effective UPR, activate autophagy, and remodel the microenvironment. Treatment can inadvertently strengthen the adaptive networks that permit residual cells to survive.

The therapeutic opportunity lies in the transition between adaptive and terminal ER stress. When the burden of unfolded proteins exceeds the capacity of the UPR to restore homeostasis, sustained PERK, IRE1α, and CHOP signaling can engage oxidative injury, mitochondrial dysfunction, and apoptosis [119]. BiP is particularly important in determining this threshold because it supports protein folding and maintains prosurvival UPR signaling. BiP depletion inhibits RCC survival under hypoxic and hypoglycemic conditions, activates CHOP-dependent apoptosis, suppresses xenograft growth, and enhances the antitumor activity of sunitinib [120,121]. Gene silencing approaches directed against BiP similarly provide proof of principle that disabling an adaptive ER chaperone can convert UPR activation into tumor-cell death. Several preclinical studies have exploited this principle pharmacologically. Temsirolimus combined with the microtubule-stabilizing agent ixabepilone synergistically reduces RCC cell viability and increases BiP and CHOP expression, implicating enhanced ER stress in the activity of the combination [122]. Histone deacetylase inhibitors have also been combined with HIV protease inhibitors to increase ER stress and kill RCC cells. Panobinostat plus nelfinavir and belinostat plus ritonavir both demonstrate enhanced preclinical activity associated with ER stress induction [123,124]. Ritonavir similarly cooperates with the proteasome inhibitor delanzomib, linking impaired protein degradation to accumulation of unresolved ER stress [125]. Sorafenib provides another example: it increases ATF4 and activates the ATF4–CHOP–PUMA pathway. Collectively, these studies support two complementary therapeutic strategies. The first is to increase the proteotoxic burden until the adaptive capacity of the tumor is exceeded. The second is to disable the pathways responsible for recovery, including chaperone function, proteasomal degradation, and autophagy. The latter strategy may be particularly effective because it prevents stress induced by the therapeutic regimen from being converted into a survival response.

Recent findings from our group place REDD1 at the center of this adaptive vulnerability. Transcriptomic analysis identified *DDIT4* as one of the most strongly induced genes in RCC cells treated with the PIM kinase inhibitor TP-3654 [126]. Pharmacologic or genetic inhibition of PIM1 activated AMPK, increased REDD1 expression, and stimulated autophagic flux. REDD1 knockout attenuated sensitivity to autophagy inhibition, whereas REDD1 overexpression increased it, establishing REDD1 as a functional determinant rather than a correlative marker of response. Chloroquine synergistically enhanced the anticancer activity of TP-3654 across RCC models, and the combination produced superior antitumor activity in vivo [126]. These findings refine how REDD1 induction should be interpreted therapeutically. TP-3654-induced REDD1 does not simply push RCC cells directly into apoptosis. It creates an autophagy-dependent state. If autophagy remains intact, this response can preserve cell viability by reducing energetic demand and recycling intracellular substrates. When autophagy is simultaneously inhibited, however, the same REDD1-driven state becomes a liability. The tumor cannot complete the adaptive program initiated in response to PIM inhibition and undergoes energetic and proteostatic collapse. In these preclinical RCC models, REDD1 functioned as a determinant of adaptive autophagy and was associated with sensitivity to autophagy inhibition. Whether REDD1 has similar predictive value in ccRCC patients remains to be determined.

The relationship between REDD1 and *VHL* further distinguishes ccRCC from other tumor types. Analysis of sporadic ccRCC demonstrated that REDD1 expression varies with *VHL* genotype. Tumors with biallelic *VHL* inactivation expressed more REDD1 than matched nonmalignant tissue, whereas tumors with monoallelic *VHL* inactivation expressed less REDD1 than wild-type or biallelically inactivated tumors [127]. REDD1 should therefore not be considered uniformly elevated in every *VHL*-altered ccRCC. Nevertheless, the association between biallelic *VHL* loss and increased REDD1 is consistent with HIF-dependent transcriptional regulation [85,127]. Both HIF1α and HIF2α can contribute to *DDIT4* induction in a cell-context-dependent manner [128]. This relationship creates a signaling paradox in ccRCC, in which HIF dependent REDD1 expression can coexist with persistent mTORC1 activity. As discussed above, this may reflect disruption or functional uncoupling of the TSC-Rheb axis through TSC1 alterations, activating mTOR mutations, reduced TSC1 dosage or other incompletely defined mechanisms [129]. This uncoupling has major biological consequences. A ccRCC cell can retain HIF- or stress-induced REDD1 while losing its ability to suppress mTORC1. Such a cell is no longer forced to choose between growth and adaptation. It can maintain anabolic signaling while preserving REDD1-dependent effects on autophagy, redox balance, and metabolic stress tolerance. This state provides a plausible explanation for why REDD1 expression can coexist with aggressive tumor behavior and why simply increasing REDD1 may not be sufficient to suppress ccRCC.

The therapeutic relevance of REDD1 therefore depends on two separable questions. First, is the downstream TSC–Rheb axis competent to translate REDD1 induction into mTORC1 inhibition? Second, does REDD1 induction create a survival dependency, particularly on autophagy, that can be therapeutically blocked? Tumors with an intact TSC complex may experience both anabolic restraint and increased autophagy following REDD1 induction. Tumors with REDD1-resistant mTORC1 signaling may experience little growth suppression but remain vulnerable to disruption of REDD1-driven stress adaptation. The most compelling strategy is therefore not indiscriminate REDD1 induction. It is deliberate induction of a REDD1-dependent adaptive state followed by blockade of the pathway required to survive in that state. More broadly, proteostasis-directed combinations can either overwhelm the ER with damaged proteins or prevent their clearance through the proteasome, p97/VCP, or autophagy. This framework transforms REDD1 from a difficult direct drug target into a functional biomarker that identifies when ccRCC cells have become dependent on stress-recovery machinery.

## 5. Therapeutic Evolution of ccRCC and the Persistence of Adaptive Resistance

Surgical resection remains the definitive treatment for localized RCC, but systemic therapy is required for unresectable or metastatic disease and for selected patients at high risk of recurrence. Historically, advanced RCC was characterized by resistance to conventional cytotoxic chemotherapy and poor clinical outcomes [130,131]. The subsequent evolution of RCC therapy has closely followed advances in its biology. Recognition of RCC as an immunogenic malignancy led to cytokine therapy; discovery of the *VHL*–HIF–VEGF axis established angiogenesis as a therapeutic target; identification of mTOR pathway activation supported development of rapalogs; structural characterization of HIF2α enabled direct HIF2α inhibition; and immune checkpoint biology transformed both metastatic and adjuvant treatment. The progression from cytokines to VEGF inhibitors, mTOR inhibitors, HIF2α inhibitors, and checkpoint blockade has produced substantial and clinically meaningful improvements. It has also revealed that ccRCC rarely remains dependent on a single pathway. The tumor can shift among angiogenic, metabolic, immune, and stress-adaptive states in response to treatment. Each major therapeutic class imposes a distinct form of stress. VEGF inhibition intensifies hypoxia and nutrient deprivation. mTOR inhibition alters protein synthesis, energetic demand, and autophagy. HIF2α inhibition disrupts metabolic and angiogenic adaptation to pseudohypoxia. Immune checkpoint therapy increases inflammatory and cytotoxic pressure within the tumor microenvironment. Cells that survive these treatments are therefore selected not only for pathway-specific resistance mutations, but also for enhanced capacity to manage ER stress, oxidative injury, and damaged proteins. This framework explains why REDD1 and proteostasis remain relevant despite the expanding therapeutic armamentarium. REDD1 can be induced by hypoxia, nutrient depletion, ER stress, ROS, and treatment exposure. Depending on downstream signaling competence, that induction can suppress mTORC1, stimulate autophagy, remodel redox homeostasis, or create a slow-growing state capable of surviving therapy. The next therapeutic advance may therefore require more than another inhibitor of a proliferative driver. It may require simultaneous targeting of the stress-recovery systems that permit tumor cells to survive otherwise effective therapy.

A compelling strategy is to pair treatments that increase the burden of damaged or misfolded proteins with agents that disable the mechanisms required for recovery. This can be achieved by interrupting the extraction of ERAD substrates through p97/VCP inhibition, preventing their degradation through proteasome inhibition, or blocking the compensatory autophagy that emerges following REDD1-mediated mTORC1 suppression. These approaches attack different components of the same adaptive system and may be particularly effective when combined with therapies that already impose metabolic, oxidative, or proteotoxic stress. The central hypothesis is that treatment resistance in ccRCC can be overcome by progressively narrowing the tumor cell’s remaining proteostatic reserve until standard-of-care therapy pushes it beyond a recoverable threshold. Within this framework, p97/VCP and proteasome inhibitors are not simply generic ER stress inducers. They are pharmacological tools for converting a reversible stress response into unresolved proteotoxic injury and, ultimately, tumor-cell death.

## 6. Targeting p97/VCP Dependent Proteostasis

p97/VCP is an abundant and highly conserved AAA+ ATPase that functions as a ubiquitin-directed protein segregase. Rather than being primarily localized to the ER, p97 is recruited through adaptor proteins to membranes, organelles, and macromolecular complexes, where it extracts or remodels ubiquitylated substrates destined for degradation or recycling [132,133]. Six p97 subunits assemble into a homohexamer containing two stacked ATPase rings, designated D1 and D2. Coordinated ATP hydrolysis generates the mechanical force required to unfold substrates and remove them from otherwise stable protein complexes. During ERAD, p97 extracts ubiquitylated proteins from the ER membrane and directs them toward proteasomal degradation, making it a critical link between recognition of defective proteins and their elimination [134,135]. This function creates a potential vulnerability in cancer cells carrying a high burden of aneuploidy, mutation, protein synthesis, and protein misfolding [135,136]. Pharmacologic inhibition of p97 interrupts ERAD, promotes the accumulation of polyubiquitylated proteins, and activates an unresolved UPR characterized by induction of BiP, XBP1s, ATF4, and CHOP [137,138]. If the accumulated damage exceeds the cell’s adaptive capacity, this response progresses to apoptosis. Importantly, the consequences of p97 inhibition extend beyond ERAD because p97 also participates in autophagy, organelle quality control, cell-cycle regulation, chromatin-associated protein turnover, and DNA-damage repair. Targeting p97 may therefore simultaneously disable several mechanisms used by tumor cells to survive proteotoxic and genotoxic stress.

CB-5083 was the first orally bioavailable, ATP-competitive p97 inhibitor to enter clinical development. CB-5083 preferentially inhibits the D2 ATPase domain and produces rapid accumulation of polyubiquitylated proteins and ERAD substrates, followed by UPR activation and apoptosis [137,138,139]. The compound demonstrated broad antitumor activity in preclinical models of multiple myeloma and several solid malignancies, including activity in xenograft models [137,138]. In xenograft models, these findings supported phase I trials in patients with advanced solid tumors and lymphoid hematologic malignancies, including multiple myeloma [140,141]. Clinical development was subsequently discontinued after dose-dependent visual disturbances, including photophobia and dyschromatopsia, were linked to off-target inhibition of phosphodiesterase-6, an essential component of retinal phototransduction [142]. Thus, the failure of CB-5083 did not invalidate p97 as an anticancer target, but it established that greater separation between p97 inhibition and retinal PDE6 activity would be required for clinical development.

CB-5339 was developed as a second-generation, orally bioavailable p97 inhibitor designed to preserve on-target potency while reducing the retinal liability of CB-5083. Relative to CB-5083, CB-5339 was approximately 15-fold less active against human cone PDE6 and achieved substantially lower retinal exposure in preclinical models while retaining potent inhibition of the p97 D2 ATPase domain [143]. CB-5339 also exhibited lower clearance and improved bioavailability across preclinical species [143]. In Acute Myeloid Leukemia (AML) models, CB-5339 produced concentration-dependent accumulation of ubiquitylated proteins and activation of ER stress. At lower concentrations, it impaired ATM signaling and homologous-recombination repair, whereas greater target inhibition produced overt proteotoxic stress. CB-5339 reduced leukemia burden and prolonged survival in syngeneic and patient-derived xenograft models and enhanced the activity of anthracycline–cytarabine chemotherapy without substantial myelosuppression in the evaluated models [143]. More recently, CB-5339 was also shown to synergistically enhance hypomethylating therapy in AML by suppressing polo-like kinase 1 (PLK1), further supporting the rationale for combining p97-directed therapy with standard of care AML treatment regimens [144]. These findings demonstrate that p97 inhibition can engage both proteostasis and DNA repair vulnerabilities rather than functioning exclusively as an ERAD inhibitor.

p97 inhibition should therefore be viewed as a mechanistically compelling strategy, but direct evidence of therapeutic dependence is currently limited in ccRCC. Its potential value may lie in preventing tumor cells from resolving the proteotoxic damage imposed by other therapies. Future preclinical studies should establish target engagement by measuring the accumulation of ubiquitylated proteins and ERAD substrates, as well as XBP1 splicing, ATF4–CHOP signaling, REDD1 induction, mTORC1 activity, and autophagic flux. These analyses will be essential to determine whether REDD1 promotes commitment to cell death or instead enables compensatory survival following p97 inhibition. Rational combinations can then be selected to intensify the initial stress burden or disable the specific recovery pathway that remains active. This framework positions p97 inhibition not as a generic ER stress inducer, but as a strategy for converting treatment-induced protein damage into irreversible proteostasis failure.

## 7. Proteasome Inhibition in Renal Cancer: Strong Biological Rationale but Limited Activity in ccRCC

The ubiquitin–proteasome system is the principal pathway through which short-lived, damaged, and misfolded intracellular proteins are eliminated. Proteins removed from the ER through ER-associated degradation are ubiquitylated and delivered to the 26S proteasome, where they are unfolded and degraded [36]. This machinery is particularly important in cancer cells burdened by aneuploidy, oncogenic protein synthesis, and mutation-associated protein misfolding [136]. Proteasome inhibition can therefore promote the accumulation of defective proteins, amplify ER stress, and activate terminal UPR signaling. However, proteasome blockade is not intrinsically lethal in every tumor. The resulting stress can also induce translational restraint, antioxidant responses, and autophagy, making the therapeutic outcome dependent on whether the adaptive response remains sufficient to restore homeostasis.

Bortezomib is a reversible boronate proteasome inhibitor that preferentially targets the chymotrypsin-like β5 catalytic subunit of the 20S proteasome [145]. It became the first proteasome inhibitor approved by the FDA following demonstration of clinical activity in multiple myeloma [146]. In ccRCC models, bortezomib inhibited proliferation and induced apoptosis independently of VHL status. Silencing p53 attenuated bortezomib-mediated growth inhibition, p21 induction, and survivin loss, indicating that p53-regulated pathways can influence sensitivity without constituting an absolute requirement for proteasome inhibition. Bortezomib also suppressed constitutive NF-κB signaling in RCC cells. Enforced expression of NF-κB components reduced apoptosis, whereas selective NF-κB inhibition alone was insufficient to reproduce the full cytotoxic effect of bortezomib. Thus, NF-κB suppression contributes to maximal apoptosis but operates together with additional proteotoxic and stress-response mechanisms [147,148].

The clinical activity of single agent bortezomib in RCC was modest. In a phase II trial, four of 37 evaluable patients achieved a partial response and 14 had stable disease, corresponding to response and disease-stabilization rates of 11% and 38%, respectively [149]. These findings demonstrated that a subset of renal cancers can respond to proteasome inhibition, but the magnitude and durability of benefit were insufficient to support monotherapy. Bortezomib also interferes with hypoxic adaptation by enhancing factor-inhibiting HIF-dependent repression of HIF1α transactivation and preventing efficient recruitment of the p300 coactivator [150]. This mechanism provided the rationale for combining bortezomib with the VEGF-neutralizing antibody bevacizumab. In a phase I study involving several advanced malignancies, 20 evaluable patients had RCC; three achieved partial responses and two maintained stable disease for at least six months [151]. Although this signal supported further investigation, the nonrandomized phase I design could not establish that the combination was synergistic or superior to VEGF-directed therapy alone.

Carfilzomib is a second-generation epoxyketone proteasome inhibitor that covalently and irreversibly binds the β5 catalytic subunit, producing more sustained proteasome inhibition than reversible boronate inhibitors [152,153]. In RCC cell lines and primary tumor cells, the HIV protease inhibitors lopinavir and nelfinavir enhanced carfilzomib cytotoxicity through mechanisms that included increased intracellular drug availability, reactive oxygen species generation, and preactivation of the UPR [154]. A separate study identified a highly specific genotype-dependent effect: bortezomib and carfilzomib stabilized the hypomorphic VHL-R167Q mutant and increased its residual capacity to suppress HIF-2α [155]. Clinical testing did not support carfilzomib monotherapy in an unselected ccRCC population [156]. Collectively, these studies show that proteasome dependence is heterogeneous across renal malignancies. The limited activity of bortezomib and the lack of responses to carfilzomib in unselected ccRCC patients suggest that basal ER stress, VHL status, or proteasome expression alone do not identify tumors likely to respond to proteasome inhibition. Differences in protein production, mTORC1 activity, redox balance, and stress response capacity may determine whether proteasome inhibition triggers apoptosis or allows tumor cells to recover. Future combinations should therefore be built around demonstrated pharmacodynamic interactions rather than the generic objective of increasing ER stress. Studies should measure proteasome inhibition, polyubiquitylated protein accumulation, ATF4–CHOP activation, REDD1 induction, mTORC1 signaling, and autophagic flux in parallel. These paired approaches would determine whether REDD1 facilitates commitment to apoptosis or reduces biosynthetic pressure and promotes recovery. It would also identify when compensatory autophagy must be inhibited to prevent escape. Because the proteasome is also required for normal protein homeostasis, treatment development must define the therapeutic window and account for toxicities. The negative monotherapy trials do not eliminate the proteasome as a therapeutic target in ccRCC; they establish that successful translation will require biomarker-selected disease, rationally sequenced combinations, and direct confirmation that the tumor’s adaptive proteostasis capacity has been exceeded.

## 8. Conclusions and Future Directions: Converting Stress Adaptation into Therapeutic Liability

ccRCC is commonly framed as a VHL–HIF-driven malignancy, but this description captures only part of its biology. VHL loss, HIF-dependent transcription, aberrant growth signaling, and metabolic reprogramming collectively impose substantial proteotoxic, oxidative, and energetic stress. Tumor survival therefore requires continuous coordination among the UPR, mTORC1, ERAD, the ubiquitin–proteasome system, and autophagy. These pathways are not merely homeostatic support systems operating in the background. They determine whether ccRCC cells survive oncogenic stress, tolerate therapy, and persist as residual disease.

REDD1 is positioned at the intersection of these adaptive responses. Under acute stress, REDD1-mediated suppression of mTORC1 can reduce protein synthesis and energetic demand, limit further cellular damage, and promote autophagic recycling. These effects may protect tumor cells until favorable conditions are restored. Under sustained or irreparable stress, however, REDD1 can contribute to oxidative injury and commitment to cell death. Its biological output also varies according to subcellular localization: cytosolic REDD1 can promote TXNIP-dependent oxidative stress, whereas REDD1 associated with mitochondria–ER contact sites can restrain mitochondrial ROS. REDD1 therefore cannot be accurately classified as uniformly tumor suppressive, oncogenic, pro-oxidant, or antioxidant. It functions as a context-dependent stress rheostat whose output is determined by the intensity and duration of stress, cellular compartment, genotype, metabolic state, and treatment exposure.

**Figure 3:**
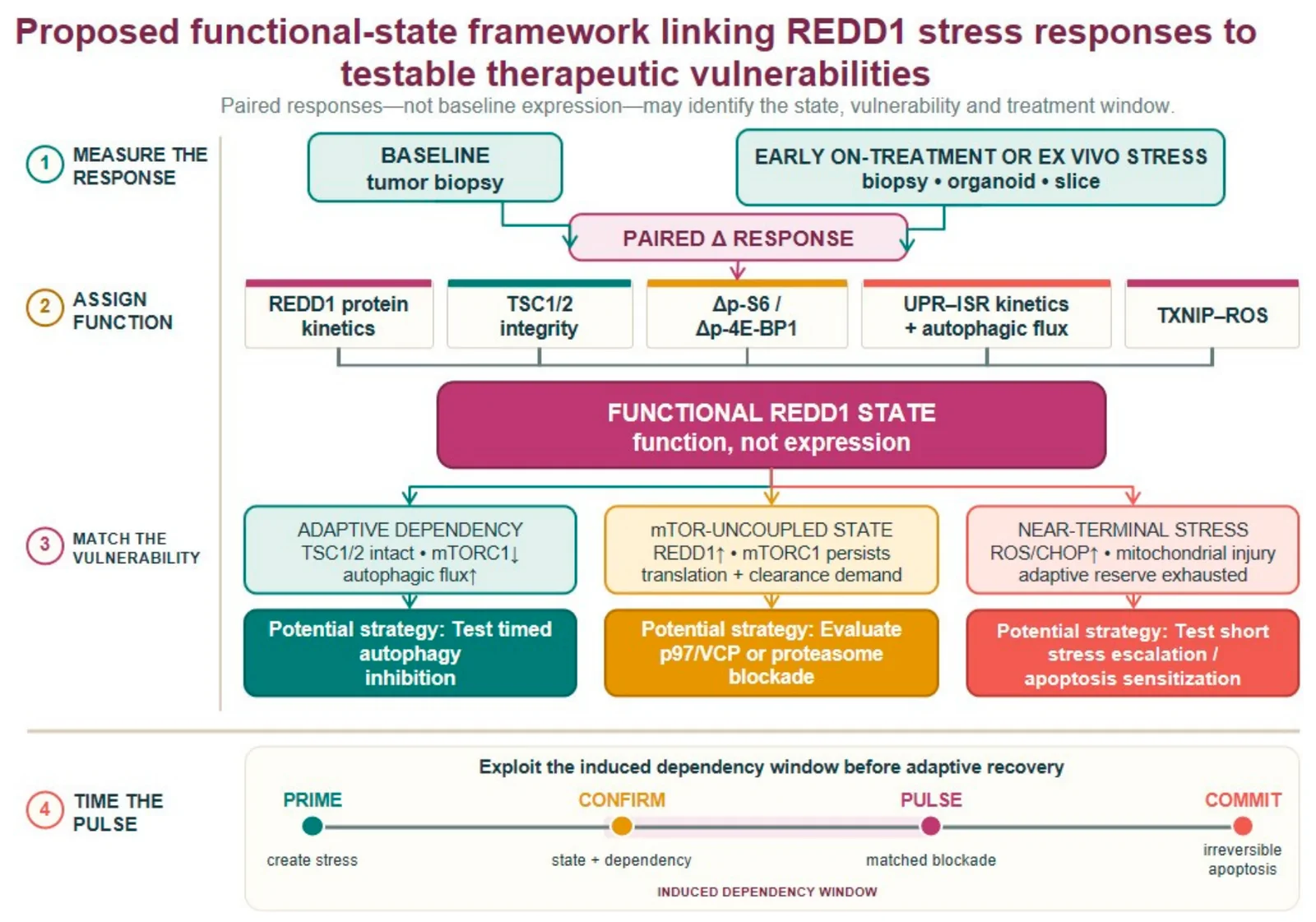
Proposed functional-state framework linking REDD1 stress responses to testable therapeutic vulnerabilities in ccRCC. This proposed framework pairs baseline tumor profiling with early on-treatment sampling or ex vivo stress testing using tumor biopsies. The paired response incorporates REDD1 protein kinetics, TSC1/2 integrity, treatment-induced changes in p-S6 and p-4E-BP1, UPR–ISR kinetics, autophagic flux, and TXNIP–ROS signaling. These integrated readouts are proposed to define a functional REDD1 state rather than relying on baseline expression. An adaptive dependency, characterized by intact TSC1/2, decreased mTORC1 activity, and increased autophagic flux, may provide a setting to test timed autophagy inhibition. An mTOR-uncoupled state, in which REDD1 increases but mTORC1 activity, translation, and clearance demand persist, may support evaluating p97/VCP or proteasome blockade. Near-terminal stress, characterized by increased ROS–CHOP signaling, mitochondrial injury, and exhaustion of adaptive reserve, may provide a setting to evaluate a short stress escalation or apoptosis sensitization. Experimental studies support the functional relationships among REDD1, mTORC1, autophagy, and stress signaling. However, using these pathways to guide treatment is still a proposed approach that needs further testing and is not yet clinically validated. This framework is hypothesis-generating and requires validation in RCC models, patient-derived systems, and prospective clinical studies before it can guide treatment selection.

This distinction fundamentally changes the therapeutic question. The objective should not be simply to induce ER stress, increase REDD1 expression, or inhibit REDD1 indiscriminately. Each of these interventions could either enhance tumor-cell killing or strengthen stress tolerance. The more compelling strategy is to determine which adaptive program REDD1 engages in a defined tumor state and then prevent that program from restoring homeostasis. In this model, standard therapies provide the initial metabolic, oxidative, or proteotoxic insult, whereas inhibition of p97/VCP, the proteasome, or compensatory autophagy closes the pathway required for recovery. The therapeutic endpoint is not stress induction itself, but irreversible failure of the stress-response network (Figure 3).

The clinical history of proteasome inhibitors in renal cancer reinforces this principle. Strong preclinical activity did not translate into meaningful benefit from unselected monotherapy in ccRCC. This failure does not establish that proteostasis is therapeutically irrelevant; it demonstrates that basal ER stress or VHL loss alone is insufficient to identify responsive tumors. The activity observed in selected molecular contexts, including SMARCB1-deficient renal medullary carcinoma and potentially hypomorphic VHL missense variants, instead suggests that proteostasis dependencies must be defined functionally and matched to the appropriate intervention with an acceptable therapeutic window. The same standard should be applied to p97/VCP inhibition. Its clinical development in ccRCC will require evidence of tumor-specific dependence, adequate target engagement, a rational combination partner, and a pharmacodynamic demonstration that adaptive recovery has been blocked (Table 2).

Several priorities will be essential for moving this field forward. First, REDD1 function must be evaluated causally using isogenic deletion, restoration, and compartment-specific rescue rather than inferred from expression alone. Second, time-resolved studies should distinguish the early adaptive effects of REDD1 from the consequences of sustained activation and determine when mTORC1 suppression and autophagy shift from protection to liability. Third, REDD1 localization, TXNIP engagement, mitochondrial redox state, mTORC1 activity, and autophagic flux should be integrated into a functional biomarker framework. *DDIT4* transcript abundance by itself is unlikely to predict therapeutic response. Fourth, these mechanisms should be tested across genetically diverse patient-derived models and in systems that preserve the tumor microenvironment. The consequences of REDD1 and proteostasis disruption for antitumor immunity are particularly important because immune-checkpoint blockade is now embedded in ccRCC treatment.

Clinical translation will likewise require dynamic measurements rather than reliance on pretreatment biomarkers alone. Paired tumor specimens should assess whether therapy produces the intended accumulation of damaged proteins, sustained ATF4–CHOP signaling, REDD1 induction, mTORC1 suppression, and compensatory autophagy. These measurements could distinguish tumors progressing because the stress pathway was never engaged from those in which stress was induced but successfully buffered. That distinction would allow combination therapy to be selected according to the active resistance mechanism rather than added empirically. However, these approaches remain preclinical. REDD1 has not been clinically validated as a predictive biomarker in ccRCC. Clinical translation will also require addressing additional factors such as tumor sampling, tumor heterogeneity, compensatory pathways, and toxicity. The central opportunity is to exploit the narrow adaptive margin under which ccRCC cells already operate. REDD1 is unlikely to represent a universal standalone target. Its greater value may be as a mechanistic determinant and dynamic biomarker of whether a stressed tumor cell recovers or dies. This possibility requires validation in clinically relevant models and prospective biomarker-guided studies. Defining that transition, and pharmacologically closing the escape route that sustains survival, could convert the stress tolerance underlying ccRCC persistence into a therapeutically actionable vulnerability.

## Figures and Tables

**Table 1:** REDD1-associated stress states and therapeutic implications.

State	Network Configuration	Dynamic Signature	Biological Interpretation	Testable Therapeutic Hypothesis
**REDD1-deficient/anabolic**	Low or weakly inducible REDD1; mTORC1 active; TSC may remain intact.	Weak REDD1 induction after stress; p-S6 and p-4E-BP1 persist; limited flux response.	The stress brake is absent. Anabolic load remains high, but buffering may be limited.	Test direct mTOR suppression or a defined proteotoxic challenge; do not assume autophagy dependence.
**REDD1-competent/adaptive**	Inducible REDD1; intact TSC1–TSC2; reversible mTORC1 suppression.	REDD1 rises, mTOR output falls, autophagic flux increases, and recovery follows.	The tumor enters a transient resource-conservation state.	Test REDD1-inducing therapy, then block autophagy during the dependency window.
**REDD1-high/mTOR-uncoupled**	High REDD1 with TSC or downstream bypass; translation remains active.	REDD1 is high with persistent mTOR output, UPR activation, and high disposal demand.	Growth suppression is bypassed while stress-adaptive outputs are retained.	Evaluate p97/VCP, proteasome, or autophagy targeting; REDD1 alone cannot select mTOR therapy.
**REDD1–TXNIP/redox-dominant**	REDD1–TXNIP signaling, thioredoxin inhibition, and elevated cytosolic ROS.	TXNIP stabilization, lower thioredoxin activity, and altered ATG4B–LC3 processing.	Redox signaling can support autophagy or amplify injury, depending on stress severity.	Test brief stress escalation or apoptosis sensitization; define normal-tissue redox liability.
**Terminal stress**	Persistent UPR–ISR, CHOP, mitochondrial injury, and failed compensation.	Protein aggregation, caspase–PARP cleavage, loss of mitochondrial potential, and no clonogenic recovery.	Adaptive capacity has been exceeded and the cell has crossed the commitment threshold.	Evaluate as a pharmacodynamic endpoint; optimize dose and schedule to cross the threshold selectively.

**Table 2:** Proteostasis-directed therapeutic landscape in renal malignancies.

Strategy/Example	Primary Node	Renal Evidence	What the Evidence Means	Critical Limitation/Next Step
**PIM inhibition + autophagy blockade**	PIM1/REDD1 induction plus lysosomal–autophagy blockade	Preclinical RCC: PIM inhibition induced REDD1 and autophagy; the combination increased apoptosis and tumor control.	Proof of principle for creating and then exploiting a REDD1-dependent vulnerability.	Use more selective autophagy agents; define sequence, biomarkers, and the normal-tissue window.
**CB-5083**	p97/VCP D2 ATPase; ERAD and protein extraction	No RCC efficacy established. Broad preclinical proteotoxic activity; oncology development ended in phase I.	p97 is pharmacologically tractable, but scaffold liability—not target biology—limited development.	Off-target visual toxicity requires next-generation selectivity and rigorous retinal safety.
**CB-5339**	Second-generation p97/VCP inhibitor	Strong hematologic preclinical activity; clinical renal efficacy remains unestablished.	Pharmacologic validation of p97 inhibition remains incomplete in renal cancer.	Establish current development status, renal pharmacodynamics, and a biomarker-defined entry strategy.
**Bortezomib**	26S proteasome	Advanced RCC phase II: partial responses in 4 of 37 patients and stable disease in 14 of 37.	A minority of tumors can respond, but unselected monotherapy is insufficient.	Identify the responder state, reduce neuropathy, and combine only with confirmed stress induction.
**Carfilzomib**	Irreversible proteasome inhibition	Refractory ccRCC phase II: no responses in 9 patients; the study stopped for futility.	Greater proteasome potency did not overcome absent selection or adaptive recovery.	Do not repeat unselected monotherapy; require functional enrichment and mechanistic scheduling.

## Data Availability

No new data were created or analyzed in this study. Data sharing is not applicable to this article.

## Abbreviations

The following abbreviations are used in this manuscript:

ccRCC	clear cell renal cell carcinoma
VHL	von Hippel–Lindau
HIF	hypoxia-inducible factor
VEGF	vascular endothelial growth factor
PDGF	platelet-derived growth factor
mTORC1	mechanistic target of rapamycin complex 1
mTORC2	mechanistic target of rapamycin complex 2
TSC1	tuberous sclerosis complex 1
TSC2	tuberous sclerosis complex 2
AMPK	AMP-activated protein kinase
ER	endoplasmic reticulum
UPR	unfolded protein response
REDD1	regulated in development and DNA damage responses 1
VCP	valosin-containing protein
ERAD	endoplasmic reticulum-associated degradation
PERK	protein kinase R-like endoplasmic reticulum kinase
IRE1α	inositol-requiring enzyme 1α
ATF6	activating transcription factor 6
ATF4	activating transcription factor 4
GRP78	glucose-regulated protein 78
BiP	binding immunoglobulin protein
XBP1	X-box binding protein 1
eIF2α	eukaryotic translation initiation factor 2α
CHOP	C/EBP homologous protein
TXNIP	thioredoxin-interacting protein
ROS	reactive oxygen species
ATM	ataxia-telangiectasia mutated
S6K1	ribosomal protein S6 kinase 1
4EBP1	eukaryotic translation initiation factor 4E-binding protein 1
PP2A	protein phosphatase 2A
Rheb	Ras homolog enriched in brain
PLK1	Polo-like kinase 1
MAMs	Mitochondria-associated endoplasmic reticulum membranes
IP3R	inositol 1,4,5-trisphosphate receptor
VDAC	voltage-dependent anion channel
